# Lithiase vésicale géante

**DOI:** 10.11604/pamj.2018.29.28.13701

**Published:** 2018-01-12

**Authors:** Yaya Ouattara, Joseph Koné

**Affiliations:** 1 Service d’Urologie, CSREF, Commune V, Bamako, Mali; 2 Service d’Anesthésie Réanimation CSREF, Commune V, Bamako, Mali

**Keywords:** Géant, lithiase vésicale, douleur, Bladder stone, giant, pain

## Abstract

A 67 years old patient has consulted for hypogastric pain, associated with a pollakiuria running for more than 12 months. The medical story reported genital prolapse, some episodes of haematuria and dysuria. Pelvic ultrasound showed a voluminous image of lithiasic appearance with irregular borders. A cystotomy was performed under perimedullary anesthesia allowing the extraction of a giant bladder stone measuring 7.2 cm as major axis and 5.5 cm for small axis.

## Image en médecine

Patiente de 67 ans ayant consulté pour douleur hypogastrique avec une pollakiurie évoluant depuis plus de 12 mois. L'interrogatoire retrouvait un prolapsus génital, des épisodes d'hématuries et dysurie. L'échographie objectivait une volumineuse formation d'allure lithiasique intra vésicale à bordures irrégulières. Une cystotomie fut réalisée sous anesthésie périmédullaire permettant l'extraction d'un calcul de forme ovoïde de 7,2 cm de grand axe et 5,5 cm de petit axe.

**Figure 1:**
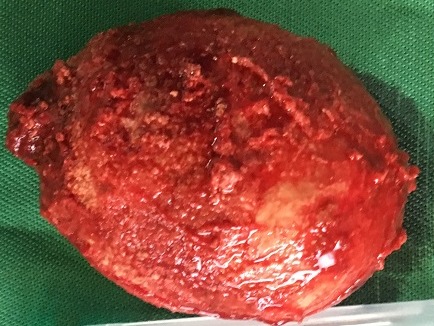
lithiase vésicale géante

